# Disulfidptosis-related genes serve as potential prognostic biomarkers and indicate tumor microenvironment characteristics and immunotherapy response in prostate cancer

**DOI:** 10.1038/s41598-024-61679-y

**Published:** 2024-06-19

**Authors:** Rongbin Zhou, Dingjin Lu, Junhao Mi, Chengbang Wang, Wenhao Lu, Zuheng Wang, Xiao Li, Chunmeng Wei, Huiyong Zhang, Jin Ji, Yifeng Zhang, Duobing Zhang, Fubo Wang

**Affiliations:** 1 Collaborative Innovation Centre of Regenerative Medicine and Medical BioResource Development and Application Co-Constructed By the Province and Ministry, Guangxi Medical University, No. 22, Shuangyong Road, Qingxiu District, Nanning, 530021 Guangxi Zhuang Autonomous Region China; 2 Center for Genomic and Personalized Medicine, Guangxi Key Laboratory for Genomic and Personalized Medicine, Guangxi Collaborative Innovation Center for Genomic and Personalized Medicine, Guangxi Medical University, Nanning, 530021 Guangxi China; 3 Department of Urology, People’s Hospital of Beihai, Beihai, 536000 Guangxi China; 4 Department of Urology, The First Affiliated Hospital of Guangxi Medical University, Guangxi Medical University, Nanning, 530021 Guangxi China; 5 School of Life Sciences, Guangxi Medical University, Nanning, 530021 Guangxi China; 6 Department of Urology, Affiliated Tumor Hospital of Guangxi Medical University, Guangxi Medical University, Nanning, 530021 Guangxi China; 7 School of Public Health, Guangxi Medical University, Nanning, 530021 Guangxi China; 8 Department of Urology, Shanghai Changhai Hospital, Naval Medical University, Shanghai, 200433 China; 9 Department of Urology, Naval Medical Center, Naval Medical Univiersiy, 338 Huaihai West Road, Shanghai, 200433 China; 10 Department of Urology, Suzhou Hospital of Anhui Medical University, 616 The Third Bianyang Road, Yongqiao District, Suzhou, 234000 Anhui China

**Keywords:** Disulfidptosis, Prostate cancer, Prognosis, Tumor microenvironment, Immunotherapy, Cancer, Computational biology and bioinformatics

## Abstract

Disulfidptosis, a newly identified programmed cell death pathway in prostate cancer (PCa), is closely associated with intracellular disulfide stress and glycolysis. This study aims to elucidate the roles of disulfidptosis-related genes (DRGs) in the pathogenesis and progression of PCa, with the goal of improving diagnostic and therapeutic approaches. We analyzed PCa datasets and normal tissue transcriptome data from TCGA, GEO, and MSKCC. Using consensus clustering analysis and LASSO regression, we developed a risk scoring model, which was validated in an independent cohort. The model's predictive accuracy was confirmed through Kaplan–Meier curves, receiver operating characteristic (ROC) curves, and nomograms. Additionally, we explored the relationship between the risk score and immune cell infiltration, and examined the tumor microenvironment and somatic mutations across different risk groups. We also investigated responses to immunotherapy and drug sensitivity. Our analysis identified two disulfidosis subtypes with significant differences in survival, immune environments, and treatment responses. According to our risk score, the high-risk group exhibited poorer progression-free survival (PFS) and higher tumor mutational burden (TMB), associated with increased immune suppression. Functional enrichment analysis linked high-risk features to key cancer pathways, including the IL-17 signaling pathway. Moreover, drug sensitivity analysis revealed varied responses to chemotherapy, suggesting the potential for disulfidosis-based personalized treatment strategies. Notably, we identified *PROK1* as a crucial prognostic marker in PCa, with its reduced expression correlating with disease progression. In summary, our study comprehensively assessed the clinical implications of DRGs in PCa progression and prognosis, offering vital insights for tailored precision medicine approaches.

## Introduction

Prostate cancer, a prevalent malignancy among men^1^, ranks as the second most common male cancer in terms of incidence. Originating primarily from the epithelium of the prostate gland, PCa is notorious for its high rates of metastasis and recurrence^2^. Despite recent advancements in the diagnosis and treatment of PCa, many patients remain at risk of post-treatment tumor recurrence and distant metastasis, contributing to a diminished five-year survival rate. Therefore, enhancing treatment efficacy is imperative. Currently, the primary treatments for PCa include surgery, radiotherapy, chemotherapy, and endocrine therapy. While these modalities can mitigate tumor progression, their therapeutic impact is limited, particularly in patients with intermediate to advanced stages of PCa or metastases. The molecular mechanisms underlying PCa development remain elusive, underscoring the need for in-depth pathogenesis research and the identification of novel biomarkers and therapeutic targets, which are crucial for advancing PCa diagnosis and treatment.

The challenge of treating prostate cancer lies in its complex molecular mechanisms and high recurrence rate, underscoring the necessity to explore new therapeutic targets. Among various treatment strategies, understanding the mechanisms of cell death is particularly crucial, especially regulated cell death (RCD)^3^, which plays a complex role in tumor control. RCD, also known as programmed cell death, includes apoptosis and various forms of regulated necrosis such as pyroptosis, autophagy, ferroptosis, and cuproptosis. These processes not only act to suppress cancer but also, in some forms, may promote tumor growth and spread. For instance, in prostate cancer, apoptosis is essential for controlling uncontrolled cell proliferation and tumor growth. Manipulating necroptotic pathways could help overcome apoptosis resistance in hormone-refractory or advanced prostate cancer, while pyroptosis is associated with adjusting immune infiltration and promoting disease development and progression.

Disulfidptosis^4^, a recently identified programmed cell death pathway, diverges from traditional mechanisms such as apoptosis and autophagy. This process is marked by excessive intracellular disulfides accumulation, exemplified by glutathione (GSH), accompanied by serine/threonine proteins' overalkylation and a decrease in the glutathione donor NADPH. SLC7A11, a crucial transporter, modulates the intracellular balance of glutathione and cysteine. Altered *SLC7A11* expression amplifies disulfide stress, leading to disulfidptosis^5^. Notably, under conditions such as glucose deprivation, tumor cells with heightened *SLC7A11* expression become more prone to disulfidptosis, characterized by aberrant disulfidation of cytoskeletal proteins like actin. This finding paves the way for leveraging DRGs as novel therapeutic avenues in PCa management.

Immunotherapy, which harnesses the immune system to identify and eliminate tumor cells, is emerging as a significant field in cancer treatment^6^. Growing evidence indicates that the levels of immune cells in tumor tissues and surrounding blood can serve as independent prognostic indicators in different cancer types. Specifically, an increase in CD8 + T-lymphocyte infiltration in PCa tissues typically indicates a more favorable immune microenvironment^7^. While advancements have been made in treating PCa with various immunotherapies^8,9^, such as checkpoint inhibitors and therapeutic tumor vaccines, the generally low sensitivity of PCa to these treatments suggests potential, yet unidentified, immune escape mechanisms.

The objective of this study is to systematically investigate the role of disulfidptosis-related genes in the biochemical recurrence of prostate cancer, as well as their efficacy in predicting immune cell infiltration, tumor mutational burden, and treatment response in prostate cancer patients. By employing bioinformatics methods and large-scale patient sample datasets, we aim to develop a risk scoring model that offers new strategies and insights for personalized treatment of prostate cancer.

## Materials and methods

### Data acquisition

We downloaded fragment per kilobase per million bases (FPKM) data, clinical data, and oncogene mutation information for 554 prostate cancer patients from The Cancer Genome Atlas (TCGA) database (https://portal.gdc.cancer.gov/). This dataset included 499 prostate tumor samples and 52 adjacent normal samples. We converted these FPKM values into transcripts per million (TPM) for standardized expression levels^9^. Additionally, we obtained FPKM values from the GEO database (https://www.ncbi.nlm.nih.gov/geo/, ID: GSE70770) and the MSKCC dataset via The Fudan Data Portal for Cancer Genomics (https://data.3steps.cn/). This included RNA-seq and clinical information on PCa, encompassing clinical T-stage, Gleason score, biochemical recurrence time, and status. These raw data were also normalized to TPM. Subsequently, we merged the mRNA expression data from the TCGA, GEO, and MSKCC datasets to form a comprehensive expression profile matrix. We applied the "Combat" algorithm to mitigate batch effects in this integrated dataset. An independent validation dataset was acquired from the GEO database (https://www.ncbi.nlm.nih.gov/geo/, ID: GSE46602). Detailed information about the DRGs is provided in Table S1.

### Consistent cluster analysis based on the expression of DRGs

We conducted an unsupervised hierarchical cluster analysis of 902 tumor samples using the R package ConsensusClusterPlus^10^. This analysis was based on mRNA expression data of 24 genes associated with disulfidptosis. The heterogeneity of the samples before and after clustering was assessed using PCA) Differences in clinical characteristics between the clusters were examined through heatmaps and box plots.

### Survival analysis, immune cell infiltration analysis, functional enrichment analysis between different subtypes

To discern survival discrepancies across clusters, we conducted survival analysis using the "Survival" package in R. Differential expression of genes (DEGs) among various disulfidptosis-related sub-clusters was identified using the limma package^11^, applying |log2 fold change|> 1 and false discovery rate (FDR) < 0.05 as thresholds for significance. Functional enrichment analysis of these DEGs was performed using the "clusterProfiler" package in R^12^, focusing on GO and KEGG pathways. We also quantified the infiltration fraction of 28 immune cells employing the single-sample gene set enrichment analysis (ssGSEA) method within the "GSVA" package^13^.

### Development and validation of a prognostic model associated with disulfidptosis

In evaluating the prognostic value of DEGs, we initially conducted univariate Cox regression analysis in the training sample set to identify DEGs linked with PFS. Excluding samples with unknown biochemical recurrence times, the remaining samples were randomly divided into a training set (n = 414) and a test set (n = 413) in a 1:1 ratio. To develop a prognostic risk scoring model for PFS and minimize the risk of model overfitting, we employed the LASSO regression method to select the most predictive DEGs^14^. The optimal lambda value was determined through this method, combined with multivariable regression analysis, leading to the establishment of the risk score model. This model computes the risk score as: risk score = ∑(Expi × Coefi), where Coefi is the risk coefficient and Expi is the expression level of gene i. Based on the median risk score, samples were classified into high-risk and low-risk groups. The prognostic potential of the risk scores was assessed using Kaplan–Meier analysis. The model's predictive accuracy was evaluated using ROC curve^15^ analysis on both the test set and the entire sample set and further validated on an independent external validation set, GSE46602.

### Construction and validation of the nomograms

In this study, we developed a nomogram utilizing DRGs risk scores to predict the prognosis of PCa. This nomogram, incorporating multiple clinical characteristics and risk scores of PCa patients, estimates 1-, 3-, and 5-year PFS probabilities based on individual patient scores. We constructed this nomogram using the "rms" package in R software. To assess the accuracy of the nomogram predictions, we evaluated them using calibration and ROC curves. Furthermore, univariate and multivariate Cox regression analyses were performed on clinical data from the Cancer Genome Atlas dataset, aiming to identify DRGs signatures and independent prognostic clinical variables in PCa, including clinical T-stage, N-stage, Gleason grading, and age.

### Tumor microenvironment and immune status in risk groups

We applied the CIBERSORT algorithm to analyze the infiltration characteristics of 22 immune cells within the tumor microenvironment^16^. Using the results from CIBERSORT, we examined immune cell enrichment in risk groups linked to disulfide bond generation. Additionally, Spearman correlation analysis was employed to investigate the relationship between risk score and immune cell infiltration. We also evaluated immune function, comparing the differences in various immune function scores between high-risk and low-risk groups. To explore the association between risk scores and response to immune checkpoint blockade (ICB) therapy, we referenced prior studies and selected 24 immune checkpoint genes for analysis^17–20^. Furthermore, we used the immunophenotype score (IPS), calculated via an unbiased machine-learning method, as an additional parameter to reflect immunogenicity, where high or low IPS indicates the precision of the results. IPS data for TCGA PRAD patients post anti-CTLA4 and anti-PD1 treatments were obtained from The Cancer Immunomics Atlas (TCIA, https://tcia.at/home), and we compared the IPS scores between the two groups^21^.

### Joint analysis of typing results and risk scores, tumor mutation, and biological function analysis

In our study, PCa patients were first classified and assigned risk scores based on findings from prior research. To validate the accuracy of these classifications and survival analysis outcomes, we employed Sankey plots and box plots as visualization methods to depict score differences across various classifications. Utilizing the "maftools" package in R^22^, we charted mutation profiles for both risk groups, displaying the frequency and types of gene mutations in the form of waterfall plots. Additionally, to elucidate the variance in TMB between the risk groups, box plots were used for visualization. We also conducted a comprehensive demonstration of the functional disparities between high- and low-risk groups through GO and KEGG analyses.

### Drug sensitivity analysis

Data on targeted drug expression and drug sensitivity in this study were sourced from the Genome for Cancer Project (GDSC, https://www.cancerrxgene.org/). The differences in sensitivity to various targeted drug treatments between high-risk and low-risk groups were analyzed using the oncopredict and parallel software packages in R^23^.

### Prognostic biomarker screening

We implemented a recursive feature elimination (RFE) method based on the random forest (RF) classifier^24,25^. This supervised machine learning technique was employed to identify genes associated with PFS prognosis. The predictive model was validated through five-fold cross-validation, and the significance of genes was assessed based on their frequency in the prognostic classifier. For the random forest screening, we set the number of decision trees to 500 and performed the screening using the randomForest package in R. Additionally, the expression levels of the *PROK1* gene were combined with clinical data from PCa patients to statistically visualize significant clinical features using box plots.

### Fluorescence quantitative PCR

Paired tumor samples of PCa tissues and adjacent non-tumor samples were obtained from the Department of Urology at the Cancer Hospital of Guangxi Medical University. Following approval from the Ethics Committee, all samples were stored at the Key Laboratory of Genome and Personalized Medicine Research of Guangxi Medical University. Total RNA extraction was conducted using the FastPure RNA Columns III kit (RC112, Vazyme, China). The extracted RNA was reverse transcribed using the HiScript III RT SuperMix for qPCR kit (R323-01, Vazyme, China). Real-time fluorescence quantitative PCR assays were performed using a LightCycler 96 system and a 2 × ChamQ Universal SYBR qPCR Master Mix kit. The PCR cycling conditions were as follows: initial denaturation at 95 °C for 30 s, followed by 40 cycles of denaturation at 95 °C for 3–10 s, annealing at 60 °C for 10–30 s, and amplification at 72 °C for 45 s, with a final extension step of 95 °C for 15 s, 60 °C for 60 s, and 95 °C for 15 s. The relative expression of the target gene was analyzed using the 2^−ΔΔCt^ method, with all primer sequences detailed in Supplementary Table S2.

### Statistical analysis

All statistical analyses in this study were conducted using R software (version 4.2.2) and GraphPad Prism 8. The Wilcoxon test was used to compare numerical differences between the two groups, with statistical significance set at p < 0.05. Results are represented with p < 0.05 denoted as *, p < 0.01 as **, and p < 0.001 as ***.

### Ethics statement

This study was conducted in accordance with the Declaration of Helsinki and was approved by the Medical Ethics Committee of Guangxi Medical University [Ethics Code 2023-KY0049].

### Informed consent

Informed consent was obtained from all subjects involved in the study. Written informed consent has been obtained from the patients to publish this paper.

## Results

### PCa and the association of 24 DRGs

**Figure 1:**
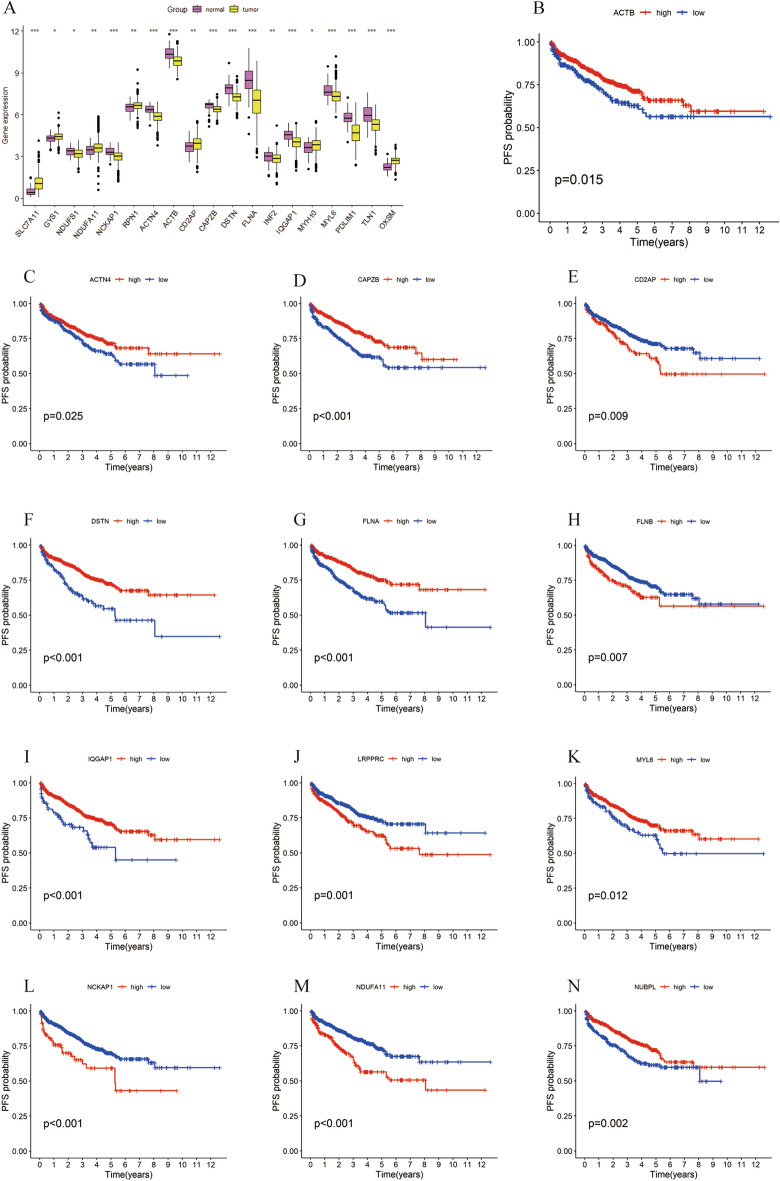
Expression of DRGs in prostate cancer patients and its association with prognosis. (**A**) Comparative analysis of DRGs expression in prostate cancer tissues and normal tissues. (**B**–**K**) Relationship between the expression level of DRGs and the probability of progression-free survival of patients.

We analyzed the expression levels of 24 DRGs in 499 prostate adenocarcinoma (PRAD) patients and 52 normal human prostate tissue samples using the TCGA-PRAD dataset. Our analysis identified differential expression of 19 DRGs between PRAD tumors and normal tissues (Fig. 1A). Kaplan–Meier survival analysis of these DRGs showed that the expression of 18 genes correlated strongly with PFS in PRAD patients. This paper presents results for 13 of these genes (Fig. 1B–N and Supplementary Table S3). High expression of *ACTB*, *ACTN4*, *CAPZB*, *DSTN*, *FLNA*, *IQGAP1*, *MYL6*, *NUBPL*, *PDLIM1*, *OXSM*, and *TLN1* correlated with improved survival. Conversely, high expression of *CD2AP*, *FLNB*, *LRPPRC*, *OXSM*, *SLC7A11*, *SLC3A2*, and *NDUFA11* correlated with shorter PFS.

### Identification of disulfidptosis subtypes and genetic subtypes

**Figure 2:**
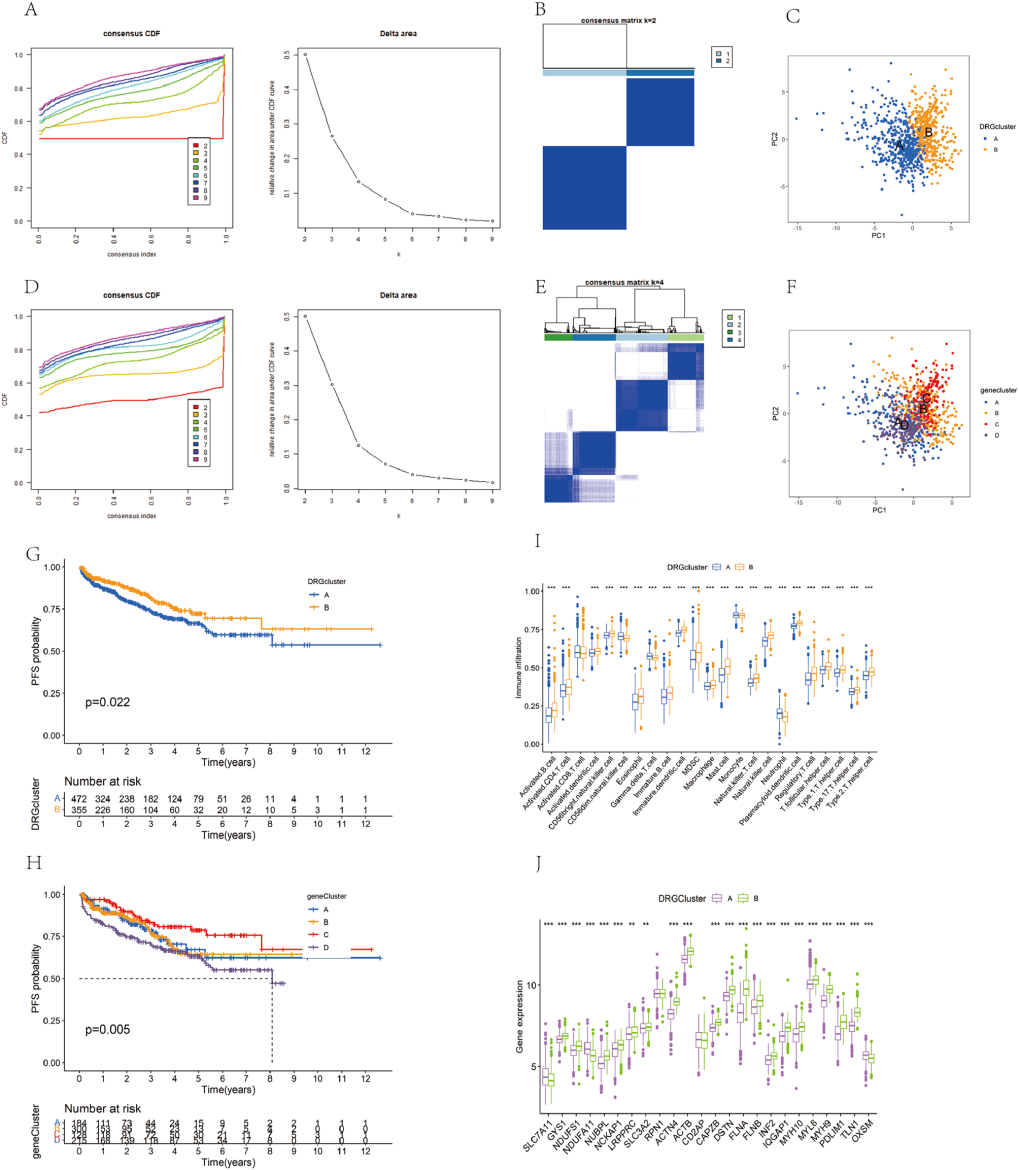
Identification and clinical correlation analysis of PCa subtypes. (**A**) CDF curves and Delta region maps show consensus clusters based on DRGs. (**B**) Heatmap of the sample concordance matrix when k = 2. (**C**) PCA analysis shows the separation of the two disulfidptosis subtypes. (**D**) CDF curves and Delta region plots based on the differential genes of the disulfidptosis subtypes. (**E**) Heat map of the concordance matrix when k = 4. (**F**) Demonstrating the distribution of different gene clusters on PCA. (**G**) Comparison of Kaplan–Meier survival curves for DRGCluster A and B. (**H**) Comparison of Kaplan–Meier survival curves for different geneClusters. (**I**) Box line plots of immune cell infiltration in different DRGCluster subtypes. (**J**) Box line plots of DRGs expression levels in different DRGCluster isoforms.

We conducted a consensus clustering analysis on 901 PCa patients from the TCGA-PRAD, GSE70770, and MSKCC datasets to investigate the association between the expression of 24 DRGs and PCa subtypes. Utilizing an unsupervised clustering approach, we varied the clustering variable k from 2 to 10, ultimately discerning two distinct regulatory modes. At k = 2 (Fig. 2A), the analysis yielded the highest intra-group similarity and lowest inter-group similarity, facilitating the classification of samples into two clusters (Fig. 2B). Subsequent downscaling analysis of PCa samples revealed significant clustering within both subtype A and B (Fig. 2C). Employing the limma package, we identified 836 differentially expressed genes (DEGs) between these subtypes. These DEGs were then used to genotype the 901 PCa samples. Examination of the cumulative distribution function (CDF) curves and Delta region maps indicated a plateau in curve progression when the cluster number was set to 4 (Fig. 2D). Further analysis using a consistency matrix (Fig. 2E) and principal component analysis (PCA) (Fig. 2F) demonstrated distinct separation among the four genetic subtypes. Kaplan–Meier survival analysis revealed that within the disulfidptosis subtypes, patients in group A had significantly better PFS than those in group B (log-rank test p-value < 0.001, Fig. 2G). Genotypically, patients with type D exhibited the shortest PFS, while those with type C had the most favorable prognosis (Fig. 2H). Additionally, we employed the ssGSEA method to assess immune cell enrichment differences between the disulfidptosis subtypes, revealing substantial variations in most immune cell infiltrations. Notably, group B showed enrichment in multiple immune cells, indicating an immunologically active state (Fig. 2I). Finally, our analysis identified distinct expressions of DRGs between the two disulfidptosis subtypes (Fig. 2J).

### Construction and validation of prognostic models

**Figure 3:**
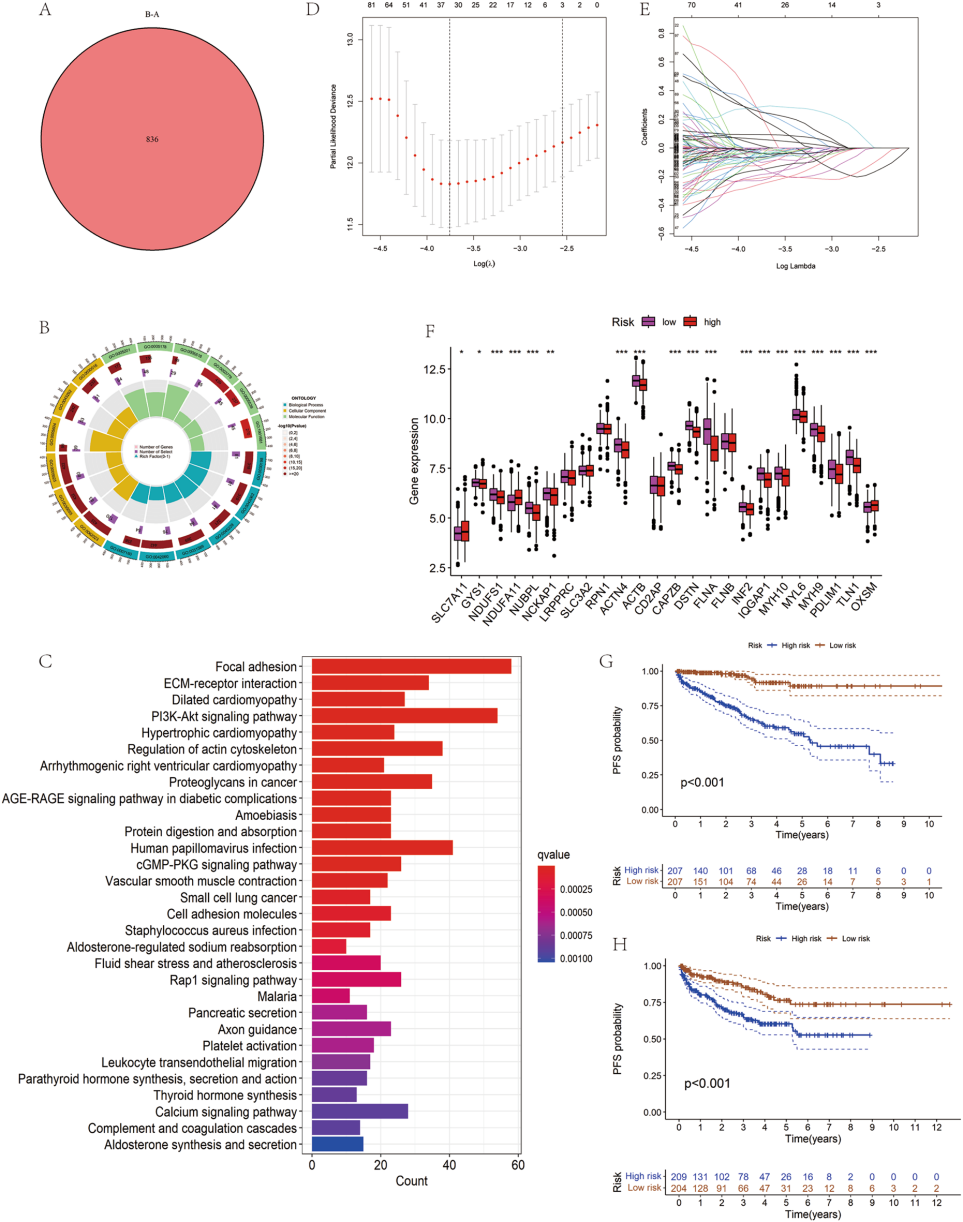
Functional enrichment and LASSO analysis of DEGs based on disulfidptosis subtypes. (**A**) Differentially expressed genes among Disulfidptosis subtypes. (**B**) String diagram showing GO and pathway functional enrichment analysis of identified DEGs. (**C**) Bar graph showing the number of DEGs involved in each KEGG pathway. (**D**) Distribution of LASSO-Cox regression coefficients for the prognostic model. (**E**) LASSO-Cox regression and ten-fold cross-validated conditioning. (**F**) Expression patterns of DEGs in patients from different risk groups. (**G**) Model training set Kaplan–Meier survival analysis. (**H**) Model validation set Kaplan–Meier survival analysis.

This study successfully identified 836 DEGs through genomic cross-analysis of two disulfidptosis subtypes (Fig. 3A; Supplementary Table S4). Utilizing one-way Cox regression analysis, we pinpointed 255 genes significantly correlated with patient prognosis (Supplementary Table S5). Functional enrichment analysis revealed that these genes were predominantly involved in an extracellular matrix organization, cell–matrix adhesion, and actin regulation in biological processes (GO-BP); in cellular components (GO-CC), they were significantly enriched in collagen's extracellular matrix, cell–matrix junction region, and myogenic fibers; for molecular functions (GO-MF), sulfide and actin-binding emerged as primary functions (Fig. 3B; Supplementary Table S6). KEGG pathway analysis showed significant enrichment in extracellular matrix receptor interactions, PI3K-Akt signalling pathway, proteoglycan synthesis in cancer, and cGMP-PKG signalling pathway (Fig. 3C; Supplementary Table S7).

Subsequently, LASSO analysis was utilized to further narrow down the pool of 255 prognostically relevant genes. The selection of the tuning parameter lambda is crucial: a smaller lambda value might include more variables, risking overfitting; conversely, a larger lambda may oversimplify the model, potentially missing critical patterns in the data. Employing LASSO regression with tenfold cross-validation (Fig. 3D), we selected the lambda value that minimized the model's cross-validation error. This approach balances model complexity with predictive performance, enhancing robustness. At this setting, 33 non-zero coefficient genes related to disulfide bond perturbation were identified (Fig. 3E). Finally, multivariate Cox regression analysis pinpointed 11 key genes, and we constructed a risk scoring model for DRGs. The formula for this DRGs risk score is: DRGs risk score = (− 0.743 × *MYADM* expression) + (− 0.617 × *MPDZ* expression) + (0.599 × *LTBP2* expression) + (− 0.320 * *PAPSS2* expression) + (− 0.352 × *DENND4C* expression) + (− 0.548 × *PROK1* expression) + (0.592 × *DDIT4* expression) + (0.579 × *IGFBP3* expression) + (− 0.381 × *CFD* expression) + (− 0.228 × *MMP7* expression) + (0.106 × *PIGY* expression). This system stratified all PCa samples into high and low-risk categories. Analyzing the expression levels of 24 DRGs between these groups, we observed significant expression differences in 19 DRGs between the high- and low-risk groups (Fig. 3F). Kaplan–Meier survival curve analysis in both test and validation sets further confirmed significantly shorter PFS in PCa patients in the high-risk DRG group compared to the low-risk group (Fig. 3G,H).To evaluate the statistical significance of the differences in progression-free survival times between high-risk and low-risk groups, we performed a log-rank test. The results of this test supported the findings from our survival curve analysis, with a *p*-value of less than 0.001, indicating a statistically significant difference in survival times between the two groups. These findings further validate our DRGs risk scoring model's effectiveness in distinguishing PCa patients with different prognoses.

### Correlation analysis between clinical characteristics, survival status, and risk scores

**Figure 4:**
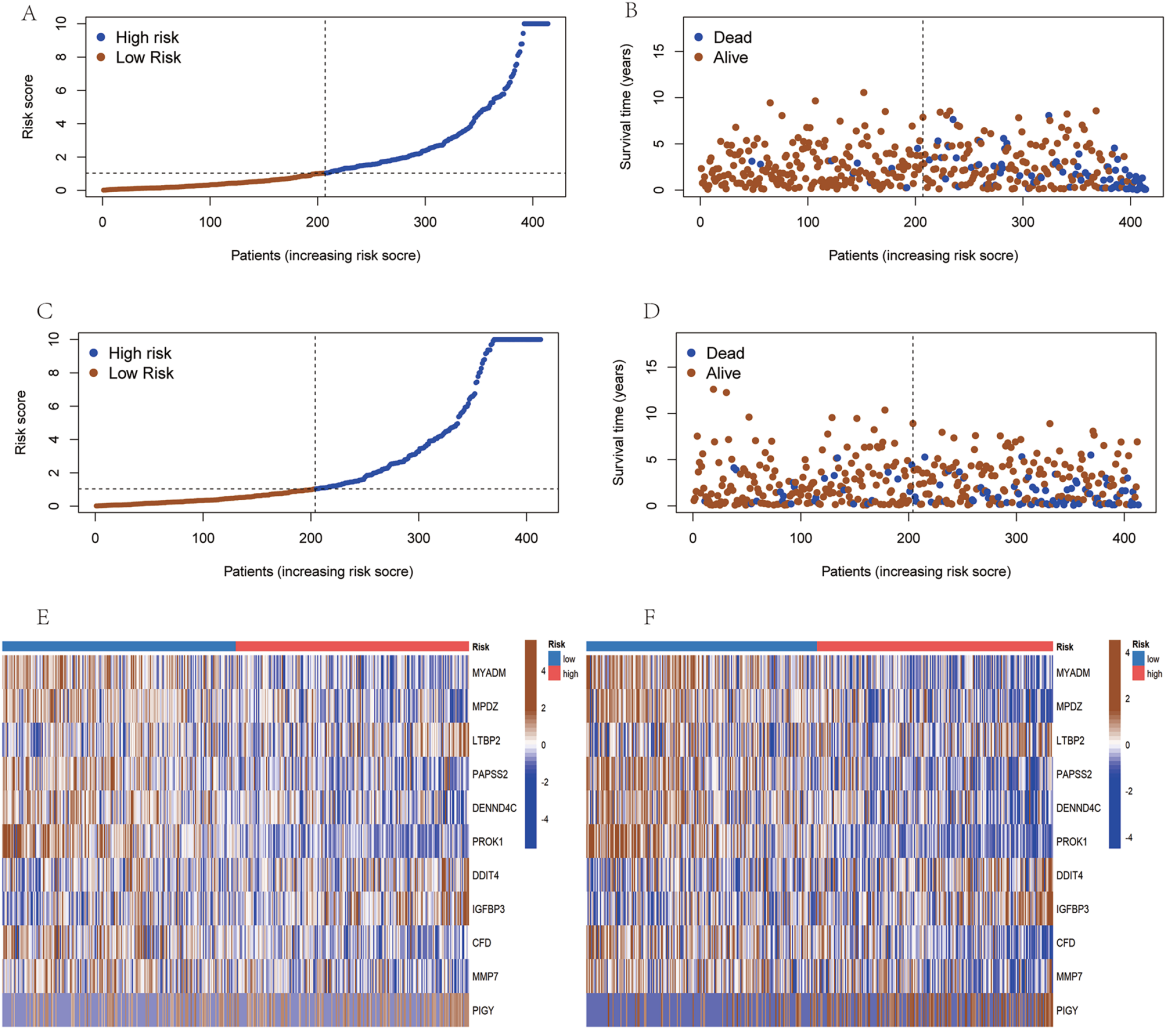
Combined analysis results of the risk assessment model for prostate cancer patients in the training, validation, and external validation cohorts (**A**–**B**–**E**) Risk profiles, survival status, and model gene expression levels of prostate cancer patients in the training cohort; (**C**–**D**–**F**) Corresponding data for patients in the test cohort.

**Figure 5.**
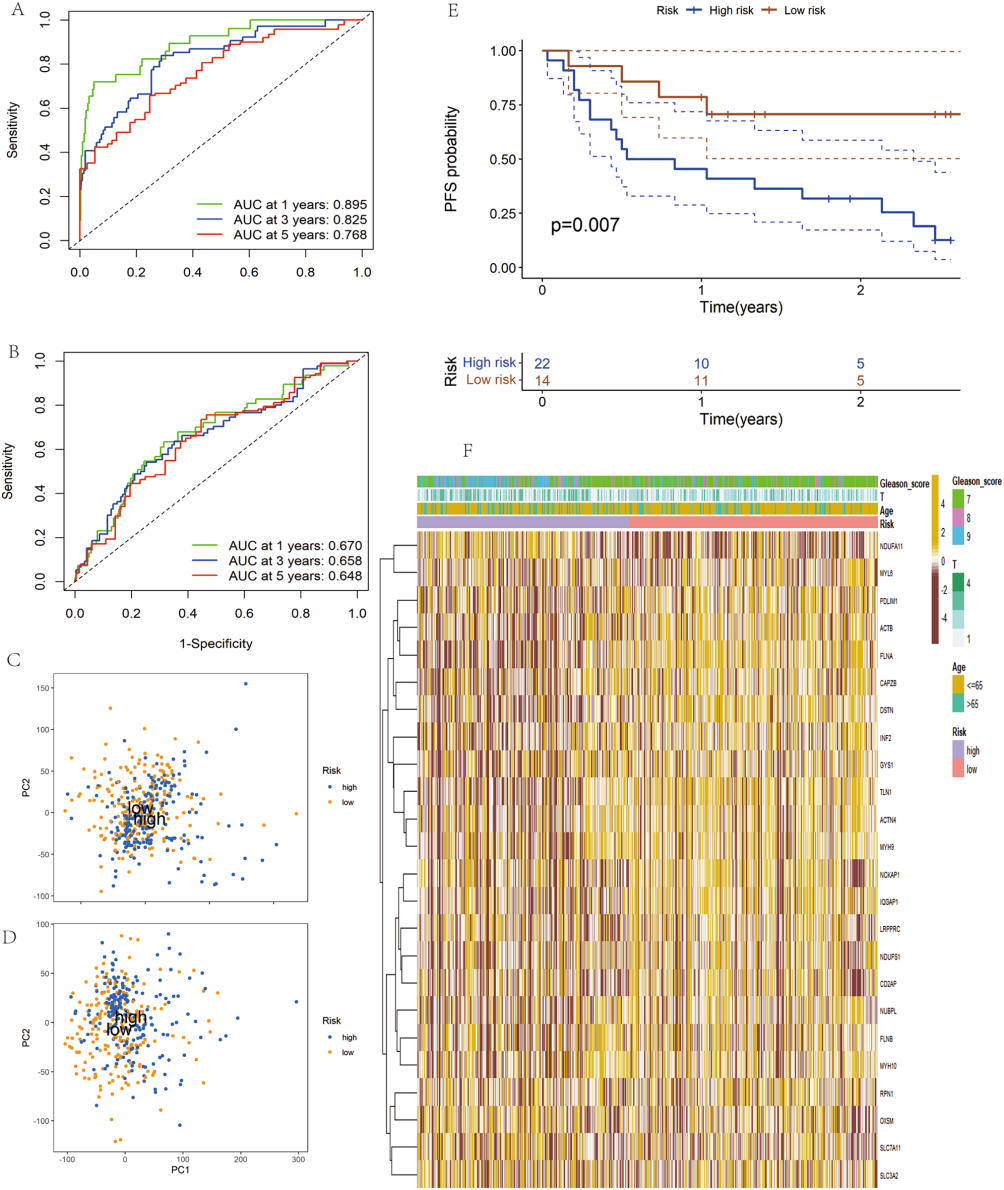
(**A**) Assessment of the accuracy of the model in predicting the prognosis of prostate cancer patients in the training cohort using ROC curves; and (**B**) The same methodology to assess the test cohort in the test cohort. (**C**) Distinguishing patients in the training set by PCA results of the DRGs risk model; (**D**) Applying the same model to distinguish patients in the test set; (**E**) Analyzing PFS survival curves of different risk groups in the external validation cohort GSE46602; and (**F**) Demonstrating heatmaps of the correlation between clinical characteristics and risk scores.

In this study, we utilized the median risk score as a threshold to delineate survival status. The results from both the training and test sets indicated that the majority of patients experiencing disease progression were in the high-risk group (Fig. 4A–D), corroborating the stability of disulfidptosis-related markers. Heatmaps demonstrated the expression patterns of eleven pivotal genes in both low and high-risk PCa patients, with consistent patterns observed in the training and test sets (Fig. 4E,F). ROC analysis results in the TCGA-train subset revealed area under the curve (AUC) values for PFS at 1, 3, and 5 years of 0.895, 0.825, and 0.768 (Fig. 5A), respectively, while in the TCGA-test subset, the corresponding AUCs were 0.670, 0.658, and 0.648 (Fig. 5B). Principal component analysis further validated the distinct separation between low-risk and high-risk PRAD patients in both test set and subset (Fig. 5C,D). To confirm these findings' validity, we extended the testing to an external validation cohort, revealing faster disease progression and poorer prognosis in high-risk PCa patients (Fig. 5E). Additionally, we investigated the correlation between risk scores and clinical characteristics, such as T-stage and Gleason scores, finding that higher T-stage and Gleason scores were associated with higher risk scores (Fig. 5F).

### Column line diagram creation, independent prognostic analysis of risk models

**Figure 6:**
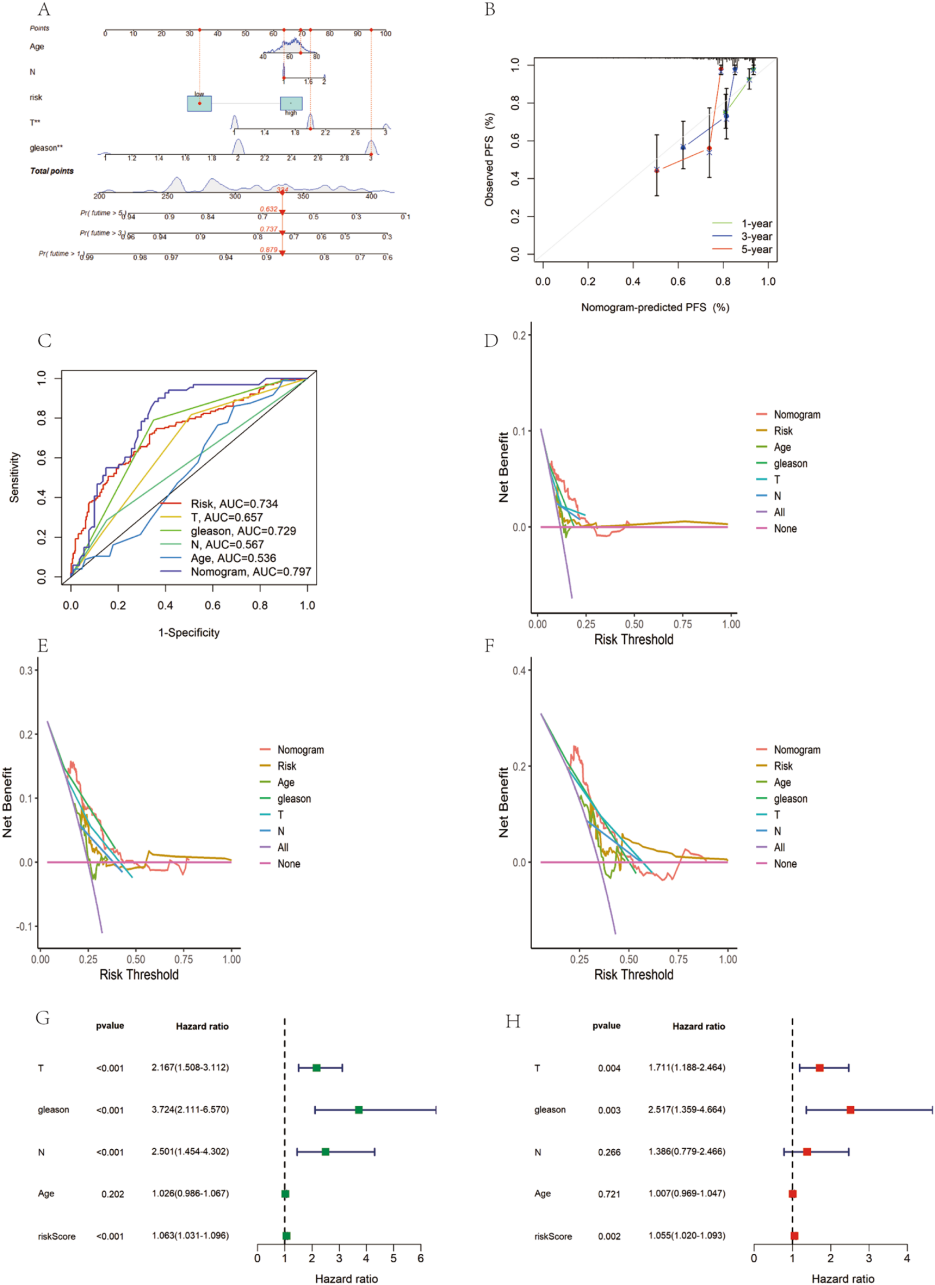
Results of independent prognostic analyses for column chart construction and prostate cancer risk modeling. (**A**) Integrated diagnostic mapping predicting PFS for prostate cancer. (**B**) Calibration curves predicting concordance with actual PFS. (**C**) AUC of model predicted PFS. (**D**–**F**) Decision curve analysis for predicting 1-, 3-, and 5-year PFS. (**G**) Results of univariate Cox regression analysis. (**H**) Results of multivariate Cox regression analysis.

We created a detailed diagnostic atlas to precisely predict PFS prognosis in prostate cancer PCa patients. This atlas specifically forecasts 1-, 3-, and 5-year PFS survival for PCa patients (Fig. 6A), incorporating essential clinical characteristics such as gender, N-staging, T-staging, Gleason score, and risk scores. Our analysis revealed a marked decline in PFS survival corresponding to increasing DRGs scores. Calibration curves demonstrated a high congruence between our model's predictions and actual observations (Fig. 6B).

In terms of predicting PFS survival, the column-line graph indicated an AUC of 0.797 for our model (Fig. 6C). Decision curve analysis (DCA) further highlighted that our diagnostic atlas most accurately predicted the 1-year PFS probability for patients (Fig. 6D–F). Additionally, both univariate and multivariate Cox regression analyses identified the DRGs score, T-staging, and Gleason score as independent prognostic indicators (Fig. 6G,H).

### Assess the role of DRGs scores in immune infiltration and immunotherapy

**Figure 7:**
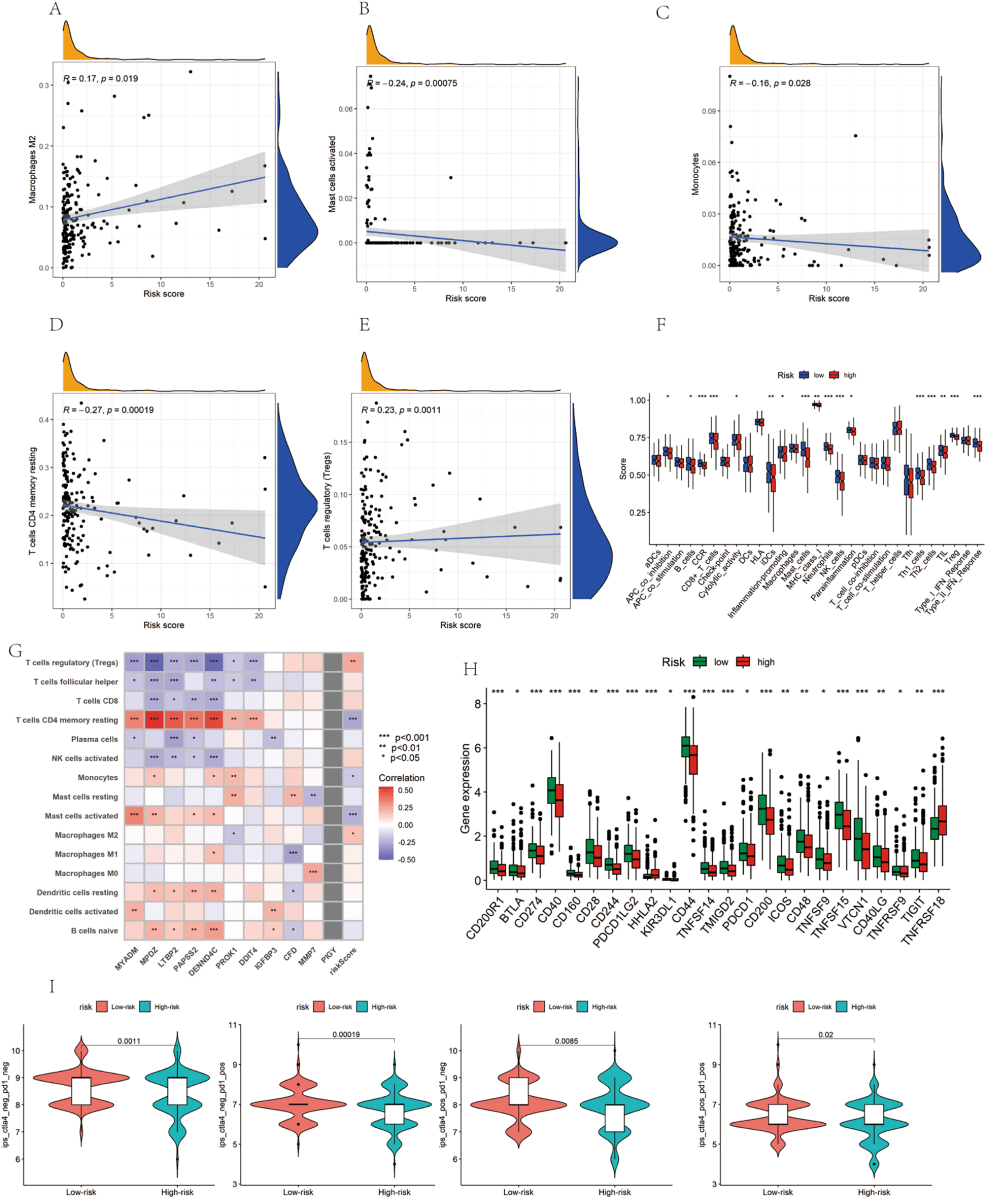
Correlation between risk scores and immune microenvironment characteristics (**A**–**E**) Correlation between risk scores and tumor microenvironment immune infiltrating cells. (**F**) Differences in tumor microenvironmental immune features across risk groups. (**G**) Heatmap presented the relationship between gene expression, risk score, and immune cell subtypes in eleven models. (**H**) Box plot showing the expression levels of immune checkpoints in different risk groups. (**I**) Differences in response to anti-PD1 and anti-CTLA-4 therapy between high and low-risk groups.

Our study initially utilized the CIBERSORT algorithm to examine the association between the DRGs risk score and immune cell infiltration. The findings indicated a positive correlation of the DRGs risk score with regulatory T cells and M2-type macrophages, while showing a negative correlation with monocytes and resting memory T cells. Furthermore, we noted a significant correlation between certain immune cells and model genes (Fig. 7A–E). In the high-risk group, there was a reduced expression of genes related to CCR, MHC class I, CD8 + T cells, NK cells, and type II IFN response, which are known to promote immune activity (Fig. 7F). A notable correlation was also observed between these eleven model genes and immune cell infiltration (Fig. 7G). In terms of immune checkpoints, crucial for immunotherapy, the low-risk group exhibited higher expression of inhibitory immune checkpoint genes *PDCD1*, *TIGIT*, and *CD274* (Fig. 7H). Additionally, we classified the patients into four categories (IPS-CTLA4-neg-PD1-neg, IPS-CTLA4-neg-PD1-pos, IPS-CTLA4-pos-PD1-neg, and IPS-CTLA4-pos-PD1-pos) (Fig. 7I) and discovered that the low-risk group had a higher immune score.

### Typing results, tumor mutation, and functional enrichment analysis

**Figure 8:**
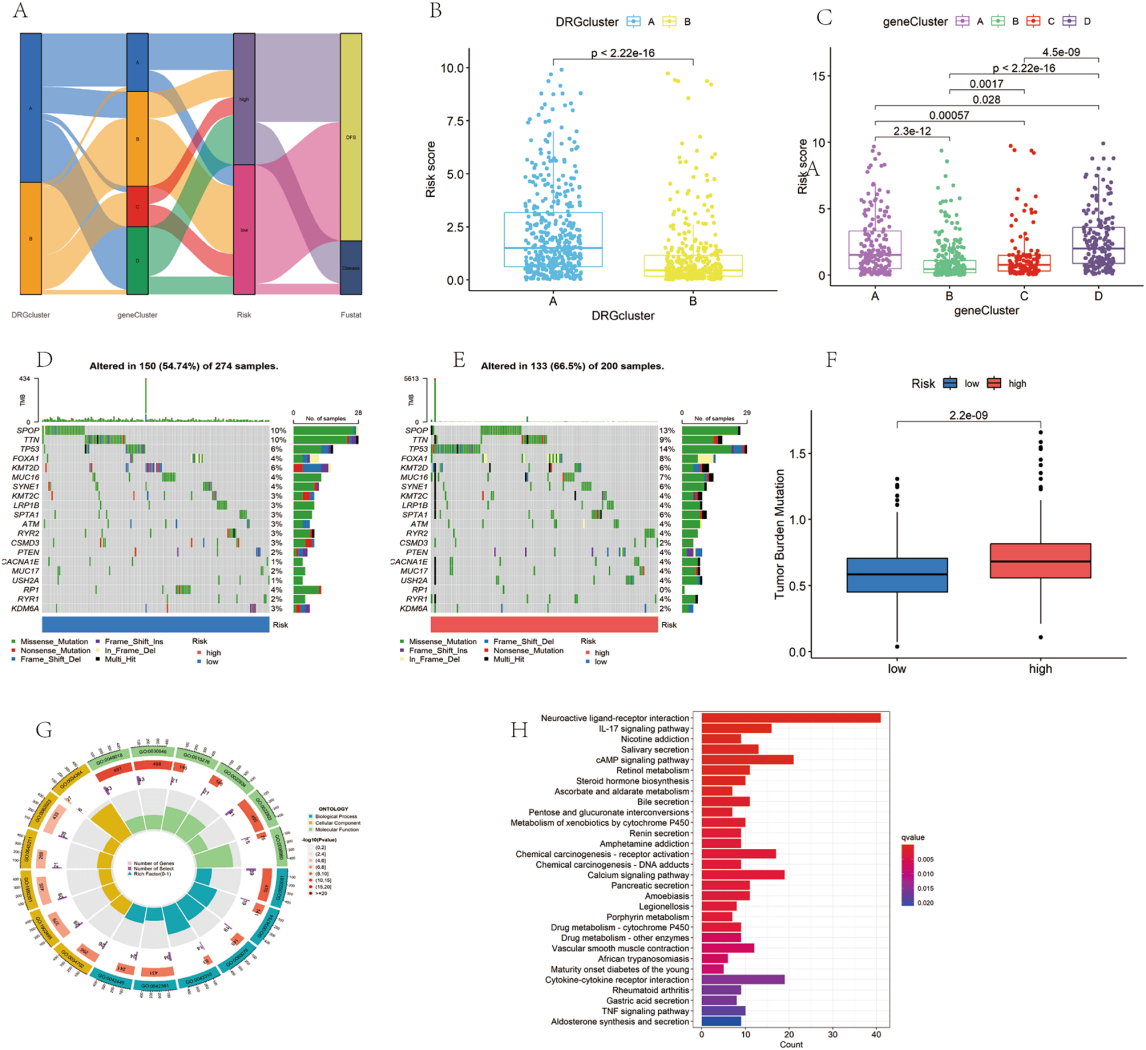
Mutation and functional enrichment analysis of tumor genes (**A**) Sankey diagram demonstrating the relationship between DRGCluster, geneCluster, risk score, and survival status. (**B**) Differences in risk ping scores between different clusters within DRGCluster. (**C**) Risk score variation between clusters within geneCluster. (**D**) Waterfall plot of gene somatic mutations in the low-risk group. (**E**) Waterfall plot of gene somatic cell mutations in the high-risk group. (**F**) Box plot depicting the difference in tumor mutation burden between low and high-risk groups. (**G**) String diagrams presenting GO pathway enrichment analysis for the high- and low-risk groups. (**H**) Bar graphs presenting KEGG pathway enrichment analysis for the high- and low-risk groups.

In our study, a Sankey diagram was used to illustrate the distribution of patients across two disulfidptosis subtypes, four genotypes, and the DRGs risk group (Fig. 8A). The results indicated that the low-risk group predominantly comprised patients with B-type subtypes, who generally did not experience significant disease progression. Further analysis showed that within the disulfidptosis subtypes, the B cluster had notably lower risk scores than the A cluster (Fig. 8B). Regarding genotypes, class B clusters exhibited the lowest risk scores compared to other clusters (Fig. 8C). Additionally, we compared mutation profiles between the high- and low-risk groups. Among the 200 patients in the high-risk group, the mutation rate was 66.5% (Fig. 8E), while it was 54.74% among the 274 patients in the low-risk group (Fig. 8D). The genes *SPOD*, *TTN*, *TP53*, *FOXA1*, and *KMT2D* showed a high mutation frequency in both groups. A relative increase in TMB was observed in the TCGA-PRAD high-risk group (Fig. 8F), potentially correlating with a better prognosis in this group.

DEGs' roles and molecular pathways in PCa in the high-risk and low-risk groups were explored using gene ontology (GO) and Kyoto Encyclopedia of Genes and Genomes (KEGG) analyses. GO analyses revealed that DEGs in the biological process (BP) category were associated with signaling release (GO:0023061), cytosolic hormone metabolic processes (GO:0034754), and vasoconstriction (GO:0042310); in the cellular component (CC) category, they were linked to the ion channel complex (GO:0034702), transporter complex (GO:1990351), and collagen-rich extracellular matrix (GO:0034364); and in molecular function (MF), they were related to receptor-ligand activity (GO:0048018), signaling receptor activation activity (GO:0030546), and ligand-gated ion channel activity (GO:0015276) (Fig. 8G, Supplementary Table S8). KEGG analysis indicated significant enrichment of DEGs in the IL-17 signaling pathway (hsa04657), neuroactive ligand-receptor interaction (hsa04080), cAMP signaling pathway (hsa04024), and steroid hormone biosynthesis (hsa00140) (Fig. 8H, Supplementary Table S9).

### Drug sensitivity analysis in low and high-risk PCa patients.

**Figure 9:**
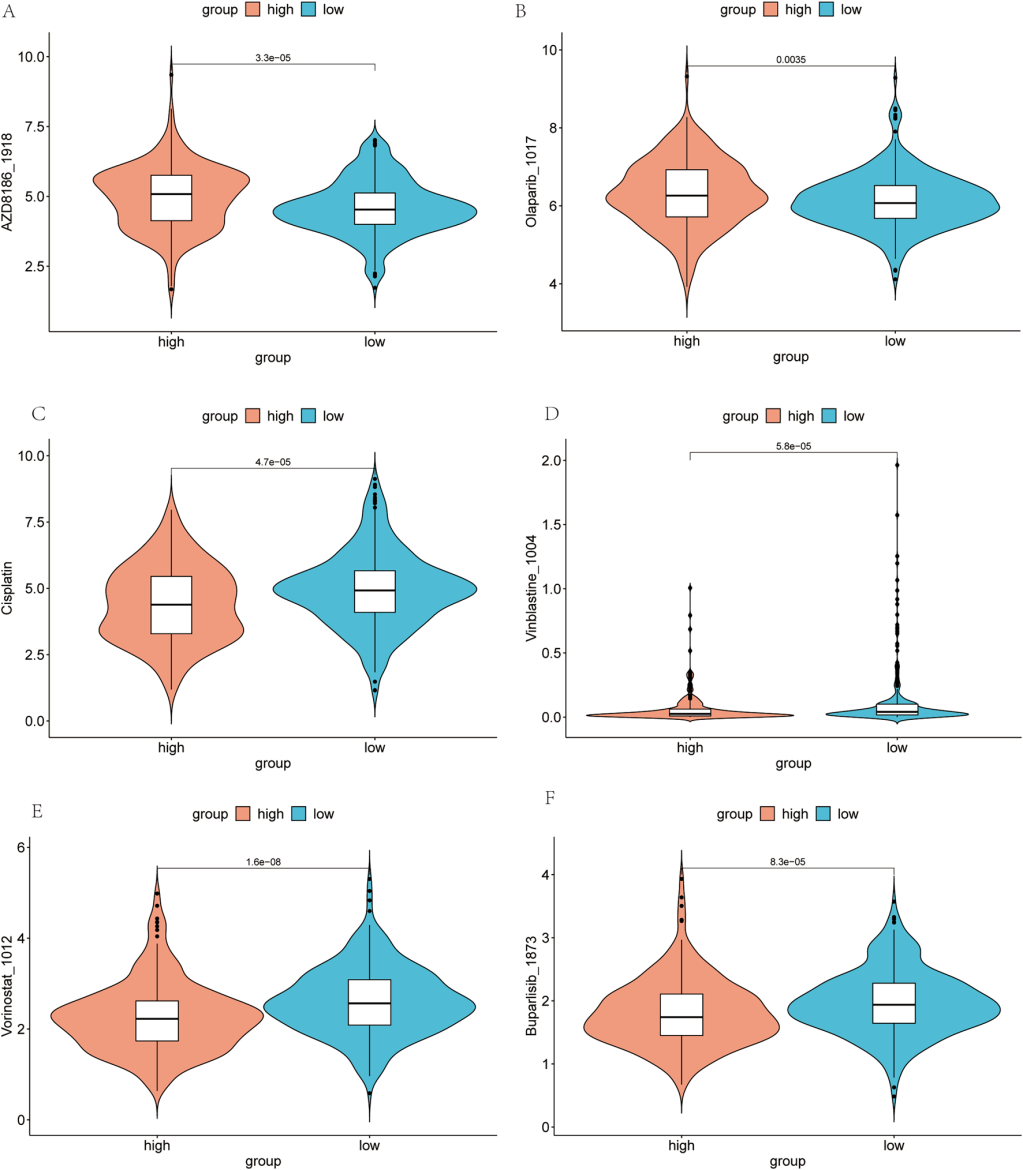
Differential analysis of drug sensitivity (**A**) AZD8186 sensitivity across low and high-risk groups. (**B**) Olaparib sensitivity across low and high-risk groups. (**C**) Cisplatin sensitivity across low and high-risk groups. (**D**) Vinblastine sensitivity across low and high-risk groups. (**E**) Vorinostat sensitivity across low and high-risk groups. (**F**) Buparlisib sensitivity across low and high-risk groups.

Through a comprehensive drug sensitivity analysis conducted on prostate cancer across varying risk levels, we constructed box plots to visually delineate the disparities among groups. The results revealed that, for patients with high-risk prostate cancer, AZD8186 exhibited a markedly enhanced sensitivity, with a median effect size significantly surpassing that of the low-risk control group (p = 6.9e−07) (Fig. 9A). Similarly, Olaparib also demonstrated a comparable trend (p = 0.00024) (Fig. 9B), suggesting that these drugs may possess improved efficacy within high-risk subgroups. On the other hand, significant differences in sensitivity between the low-risk and high-risk groups were observed for Cisplatin (Fig. 9C), Vinblastine (Fig. 9D), Vorinostat (Fig. 9E), and Buparlisib (Fig. 9F). This was evident from the overlapping interquartile ranges and median values, with the p-value outcomes (Cisplatin p = 0.00036; Vinblastine p = 0.00016; Vorinostatp = 6.8e−08; Buparlisib p = 0.00041) further substantiating the statistical significance of these disparities.

### *PROK1* may be an essential prognostic, predictive marker in PRAD

**Figure 10:**
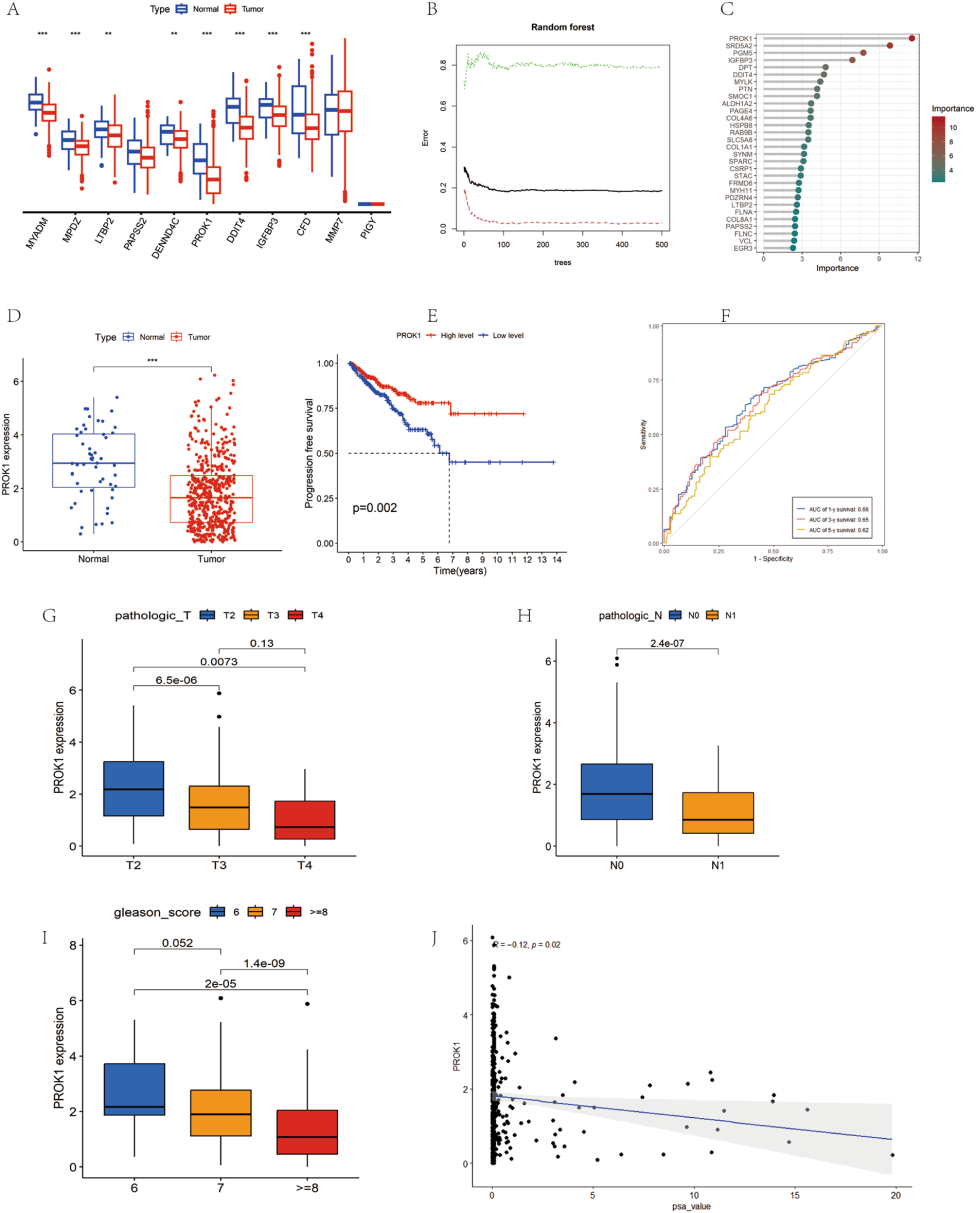
Biomarker screening (**A**) Differential expression of eleven model genes in normal tissues and tumors. (**B**) Importance of prognosis-related genes determined by the randomized survival forest algorithm. (**C**) *PROK1* has the highest importance among all prognosis-related genes. (**D**) *PROK1* expression in normal tissues and PRAD tumors. (**E**) Relationship between *PROK1* expression levels and PFS in PCa patients. (**F**) ROC curve of *PROK1* in the prognostic prediction of PCa patients. (**G**) Relationship between *PROK1* expression and T stage. (**H**) Correlation between *PROK1* and N staging. (**I**) Correlation between *PROK1* expression and Gleason score. (**J**) Negative correlation between *PROK1* expression and PSA level.

Upon analyzing the expression of 11 model genes in prostate PRAD, we observed significant differential expression of eight genes (*MYADM*, *MPDZ*, *LTBP2*, *DENND4C*, *PROK1*, *DDIT4*, *IGFBP3*, and *CFD*) between normal and tumor tissues (Fig. 10A). We then assessed the relative significance of 255 prognostically relevant DEGs using the random survival forest (RSF) algorithm, finding *PROK1* to exhibit the highest prognostic efficacy (Fig. 10B,C). Further investigations revealed that *PROK1* expression was down-regulated in PCa (Fig. 10D), with low *PROK1* expression associated with poorer PFS (Fig. 10E). The area under the ROC curve for predicting the 1-year prognosis of PCa patients based on *PROK1* expression was 0.66 (Fig. 10F). *PROK1* expression was notably lower in PCa patients with higher T-stage, N-stage, and Gleason scores (Fig. 10G–I) and was inversely correlated with prostate-specific antigen (PSA) levels (Fig. 10J).

### Validation in PCa patients

**Figure 11:**
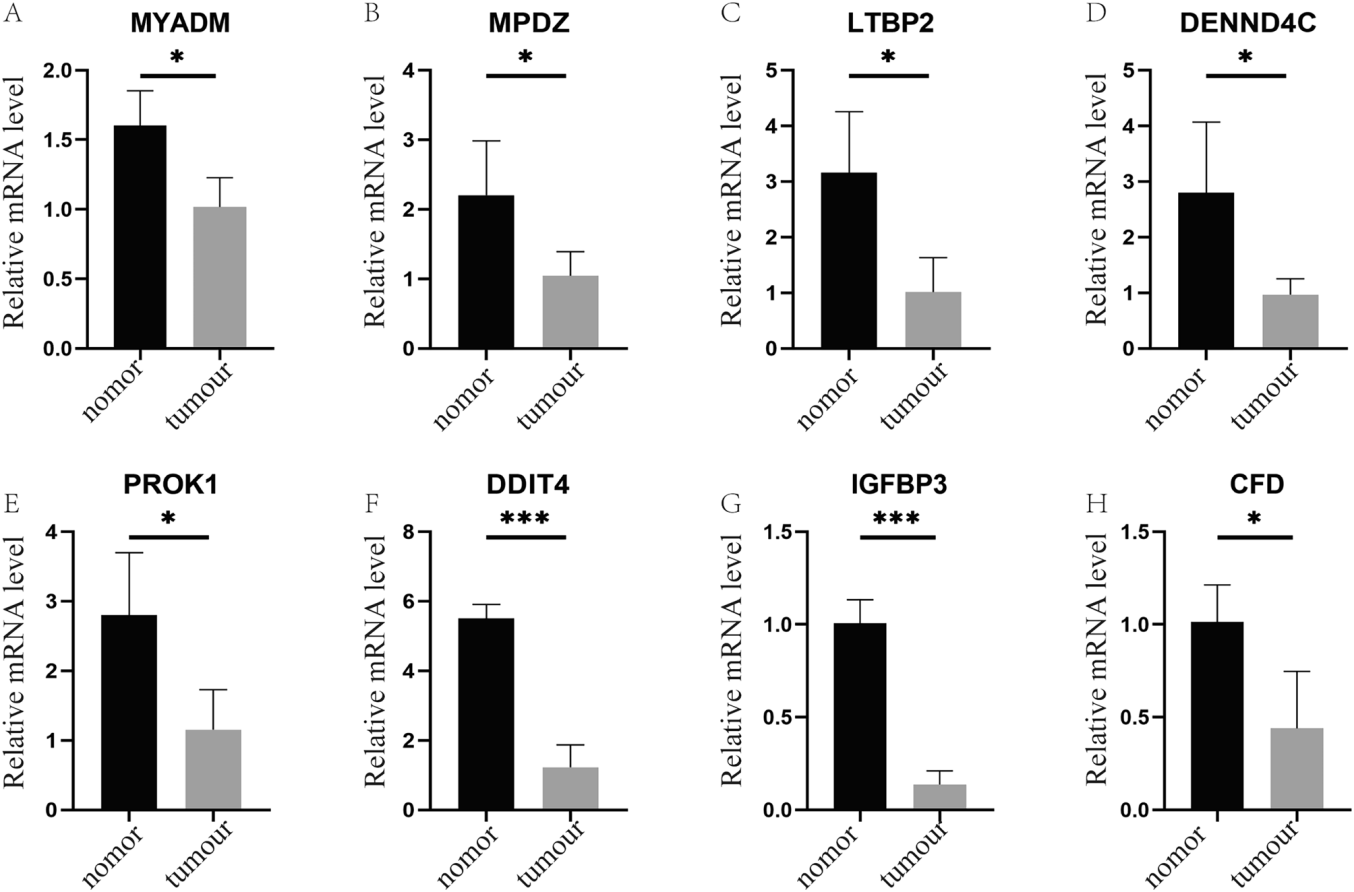
RT-qPCR validation (**A**–**H**) The mRNAs of *MYADM*, *MPDZ*, *LTBP2*, *DENND4C*, *PROK1*, *DDIT4*, *IGFBP3*, and *CFD* were significantly down-regulated in prostate cancer tissues compared to normal tissues.

In this study, we collected a range of PCa tissues and corresponding normal tissue samples from Guangxi Medical University Cancer Hospital. Utilizing RT-qPCR technology, we examined the mRNA expression levels of a specific set of differential genes. Our findings revealed that the mRNA expression levels of *MYADM*, *MPDZ*, *LTBP2*, *DENND4C*, *PROK1*, *DDIT4*, *IGFBP3*, and *CFD* were significantly down-regulated in PCa tissues compared to normal tissues (Fig. 11A–H). These results align with our preceding bioinformatics analysis, further corroborating the expression patterns of these genes in PCa development.

## Discussion

Prostate cancer, a significant global men's health concern, has seen a rising incidence in recent years^26^. The conventional treatment modalities for PCa, primarily hormone therapy, radiotherapy, and chemotherapy, often encounter resistance in patients with desmoplasia-resistant prostate cancer, resulting in poor prognosis and high recurrence rates^27^. This situation underscores the urgent need for novel therapeutic strategies and the criticality of personalized treatment approaches in clinical settings, particularly for older patients with compromised overall health. The emergence of immunotherapies and targeted therapies represents promising new treatment avenues^28^. However, the effectiveness of these therapies hinges on accurately identifying patient populations that are most likely to benefit from them. In this study, we focused on disulfidptosis, as proposed by Liu, offering a novel perspective on the role of disulfide bonds and altered glucose metabolism in cell death. The involvement of DRGs in prostate tumorigenesis remains uncertain. This study sheds light on the significance of disulfidptosis in PCa, particularly in predicting patient prognosis, recurrence status, responsiveness to immunotherapy, and drug sensitivity. Our findings may contribute to identifying pivotal biomarkers that enhance the clinical diagnosis and treatment of PCa.

In this study, we first conducted an extensive genetic and transcriptional analysis of DRGs in PCa. The findings revealed significant expression differences for most DRGs between tumor and control samples. From the expression patterns of these DRGs, we identified two molecular clusters associated with disulfidptosis. Notably, patients classified under subtype B PCa exhibited enhanced PFS and increased levels of immune cell infiltration, aligning with prior research in PCa. This discovery underscores the importance of molecular typing in predicting PCa patient prognosis and their responsiveness to neoadjuvant chemotherapy and immune checkpoint inhibitor therapy^29^.

To further explore the role of disulfidosis markers in PCa, we identified 11 key genes from 255 prognostically relevant differentially expressed genes using Lasso-Cox regression analysis. A risk scoring model was developed based on these eleven key genes. Internal and external validations further demonstrate the robustness and predictive accuracy of these prognostic markers. MYADM is notably overexpressed in African American men who experience biochemical recurrence within 5 years^30^. Zhang et al. suggest that LTBP2 is not only a promising diagnostic biomarker for biochemical recurrence of prostate cancer but also plays a significant role in regulating the immune microenvironment, affecting responses to immunotherapy, and inhibiting cancer cell proliferation and metastasis through the PI3K/AKT signaling pathway^31^. PAPSS2, a gene associated with androgen synthesis, is proposed by Ibeawuchi et al. to influence the progression of prostate cancer, where its gene deletion is closely linked to an increased risk of postoperative PSA recurrence, positioning PAPSS2 as a potential predictive biomarker for PSA recurrence^32^. Pasquali et al. propose that PROK1 may play a crucial role in the development of prostate cancer, particularly through its regulation of angiogenesis^33^. DDIT4, identified as a novel EMT-related gene by Zhao, is regulated by m6A and plays a critical role in promoting EMT, motility, and invasive metastasis in prostate cancer cells^34^. Chen et al. report that high expression of IGFBP3 in castration-resistant prostate cancer is closely associated with disease progression, and LOX can inhibit the development of castration-resistant prostate cancer by suppressing IGFBP3 expression^35^. CFD, a key component of the complement cascade, is suggested by Loveridge et al. to influence the tumor immune microenvironment and participate in BRF1-mediated prostate carcinogenesis^36^. Zhang et al. highlight MMP7 as not only a key promoter of EMT in prostate cancer but also an important downstream effector molecule of IL-17's oncogenic effects. Other genes have not yet been reported in prostate cancer^37^.

Enrichment analysis in our study indicated that differential gene expression in high-risk patients was significantly linked with the IL-17 signaling pathway and the cAMP signaling pathway. Notably, cytokines of the IL-17 family are known to foster tumor angiogenesis and cell proliferation^38,39^. Concurrently, the cAMP signaling pathway plays a vital role in regulating cell proliferation and differentiation^40^. Aberrant activation of these pathways has been closely linked with the development of various cancers. Prior research, including studies by Song et al., Mousa et al., and Zhang et al., has demonstrated IL-17's role in promoting the progression of numerous cancers, such as PCa, colon, skin, breast, lung, and pancreatic cancers^41,42^. For instance, Song et al. highlighted that IL-17, produced in the tumor microenvironment, supports tumor cell survival by stimulating angiogenesis. Additionally, Mousa et al. identified IL-17 as a significant predictor of disease progression or recurrence in bladder cancer^43^. Zhang et al.'s study further elucidated IL-17's contribution to enhancing epithelial-mesenchymal transition (EMT) and increasing prostate tumor invasiveness^37^. This evidence may account for the poorer prognosis observed in high-risk patients, suggesting that the activation of IL-17 and cAMP signaling pathways could be linked to the worsened condition of these patients.

Subsequently, we assessed the efficacy of our model in predicting PFS in PCa patients using ROC analysis. In the TCGA-train subset, the model exhibited robust performance, predicting 1-, 3-, and 5-year PFS with AUC values of 0.895, 0.825, and 0.768, respectively. However, in the TCGA-test subset, there was a slight decrease in predictive performance, with AUCs of 0.670, 0.658, and 0.648 for 1-, 3-, and 5-year PFS, respectively. Additionally, using the column-line graph for PFS survival prediction, the model showed good predictive capability, with an AUC of 0.797. These findings indicate that while the model's performance varied across different subsets, it generally exhibited good accuracy and stability in predicting PFS in PCa patients. Consequently, this model holds potential for application in assessing biochemical recurrence in PCa patients.

We conducted an immune infiltration analysis to investigate disulfidptosis in the tumor immune microenvironment. Our results reinforced the critical role of the tumor immune microenvironment in tumor development, progression, and treatment resistance. Analyzing the correlation between risk scores and the proportion of regulatory T cells (Tregs), we found that high-risk scores were markedly associated with an increase in Tregs. This aligns with existing research on Tregs' role in the tumor microenvironment, where their proliferation is often seen as a pivotal factor in tumor immune escape^44^, promoting tumor evasion and worsening prognosis by inhibiting the host's anti-tumor immune response. Our data indicate that a higher proportion of Tregs might correlate with immune escape and poorer prognosis in high-risk patients. Furthermore, our study differentiated the distinct roles of M1-type and M2-type macrophages in tumor progression. M1-type macrophages, with their proinflammatory effects^45^, can activate immune responses, potentially hindering tumor progression. In contrast, M2-type macrophages facilitate tumor cell proliferation^46^, angiogenesis, and metastasis by secreting pro-growth and pro-angiogenic factors, as well as elastase. The increased infiltration of M2-type macrophages in high-risk groups could account for the accelerated tumor progression and poorer patient outcomes. These findings offer new perspectives on the immunological mechanisms driving poor prognosis in high-risk groups.

Next, our study observed that four key immunosuppressive checkpoints (*PDCD1*, *CTLA4*, *CD274*, and *TIGIT*) were significantly upregulated in low-risk PCa patients. This finding indicates potential distinct immune escape mechanisms in low-risk patients compared to other groups. *CTLA4*, known for suppressing activated T cells and enhancing regulatory T cells (Treg) function^47,48^, and PD-1, critical in tumor immune escape by inhibiting immune cell activity via binding to tumor cell surface ligands^49^, suggest a relatively active tumor microenvironment in low-risk patients. This environment might be more amenable to immune checkpoint inhibitor therapy. Further, immunopredictive score (IPS) analysis revealed notable differences in response to PD1 and CTLA4 inhibitors between high-risk and low-risk groups. Generally, higher IPS scores indicated a better response to PD-1 or CTLA-4 therapy, implying that tumors in low-risk patients are more responsive to immune checkpoint inhibitor therapy. These insights offer potential biomarkers for evaluating immunotherapy response and provide a crucial foundation for risk-based stratification and the implementation of personalized immunotherapy strategies.

Applying the randomized survival forest algorithm, we identified *PROK1* as a key gene in PCa, offering fresh insights into its molecular mechanisms. Our findings demonstrated that low expression of *PROK1* in PCa is significantly linked to poor prognosis^33^, aligning with its role in other malignancies such as pancreatic ductal carcinoma and neuroblastoma^50–52^, where *PROK1* expression correlates with tumor progression and metastasis. This correlation between *PROK1's* low expression and adverse outcomes in PCa underscores its critical role in tumor development. Previous research indicates PROK1's pivotal role in tumorigenesis and progression, potentially impacting tumor malignancy. For instance, in colorectal cancer^53^, *PROK1* and *VEGF* co-expression was strongly associated with poorer prognosis, more pronounced lymph node and hematogenous metastasis, and advanced TMN stage disease. These results imply that *PROK1* may regulate the tumor microenvironment and facilitate tumor metastasis.

By comparing studies on prostate cancer, sarcoma^54^, renal cell carcinoma^55^, and cervical cancer^56^, we discovered that the expression of disulfidptosis-related long non-coding RNAs and genes is closely linked to tumor prognosis^57^. These studies collectively highlight the critical role of disulfidptosis-related genes in tumor development and progression, and their significant correlation with patient prognosis, tumor microenvironment remodeling, and immune responses. The constructed risk scoring model can guide prognostic assessments and personalized treatment strategies for patients.

While this retrospective study has yielded valuable findings, there are limitations that need addressing. Firstly, the data utilized in this study are derived solely from public databases and lack prospective validation data from multicentric clinical cohorts. This undoubtedly limits the assessment of our model's applicability to a broader population. Thus, future studies should ideally be based on multicentric prospective cohorts, which would help reduce bias and improve data quality, thereby enhancing the translatability and generalizability of our findings in clinical practice.

## Conclusions

In this research, we conducted the first systematic analysis of DRGs and transcriptomic alterations in PCa. By developing a risk prediction model incorporating 11 key features, we identified notable differences in the tumor immune microenvironment, IL-17 signaling pathway, and cAMP signaling pathway between high and low-risk PCa patient groups. These differences were strongly linked to patient prognosis, underscoring the model's robust ability to predict both prognosis and response to immunotherapy in PCa patients. Our findings introduce novel biomarkers for prognosticating clinical outcomes in PCa patients and establish a theoretical basis for advancing immunotherapy and personalized treatment approaches for PCa. This study is pivotal in enhancing our understanding of PCa pathogenesis and guiding the development of innovative therapeutic strategies.

### Supplementary Information

Supplementary Figures.

Supplementary Information.

## Data Availability

The datasets supporting the conclusions of this study are derived from several publicly accessible repositories, ensuring transparency and reproducibility. Specifically, The Cancer Genome Atlas (TCGA): Available at https://portal.gdc.cancer.gov/.GSE70770: Available at https://www.ncbi.nlm.nih.gov/geo/query/acc.cgi?acc=GSE70770.The MSKCC dataset: Available at https://data.3steps.cn/.GSE46602: Available at https://www.ncbi.nlm.nih.gov/geo/query/acc.cgi?acc=GSE46602.

## Code availability

The R code utilized for data analysis and figure generation is available on GitHub at https://github.com/good1000/123.
