# Supplementary material for: Disulfidptosis-related genes serve as potential prognostic biomarkers and indicate tumor microenvironment characteristics and immunotherapy response in prostate cancer
Source: Sci Rep. 2024 Jun 19;14:14107. doi: 10.1038/s41598-024-61679-y (PMC11187134; doi:10.1038/s41598-024-61679-y)
Supplement: Supplementary file 1 — Supplementary Figures. [file 41598_2024_61679_MOESM1_ESM.pdf]

Supplementary Figure 1

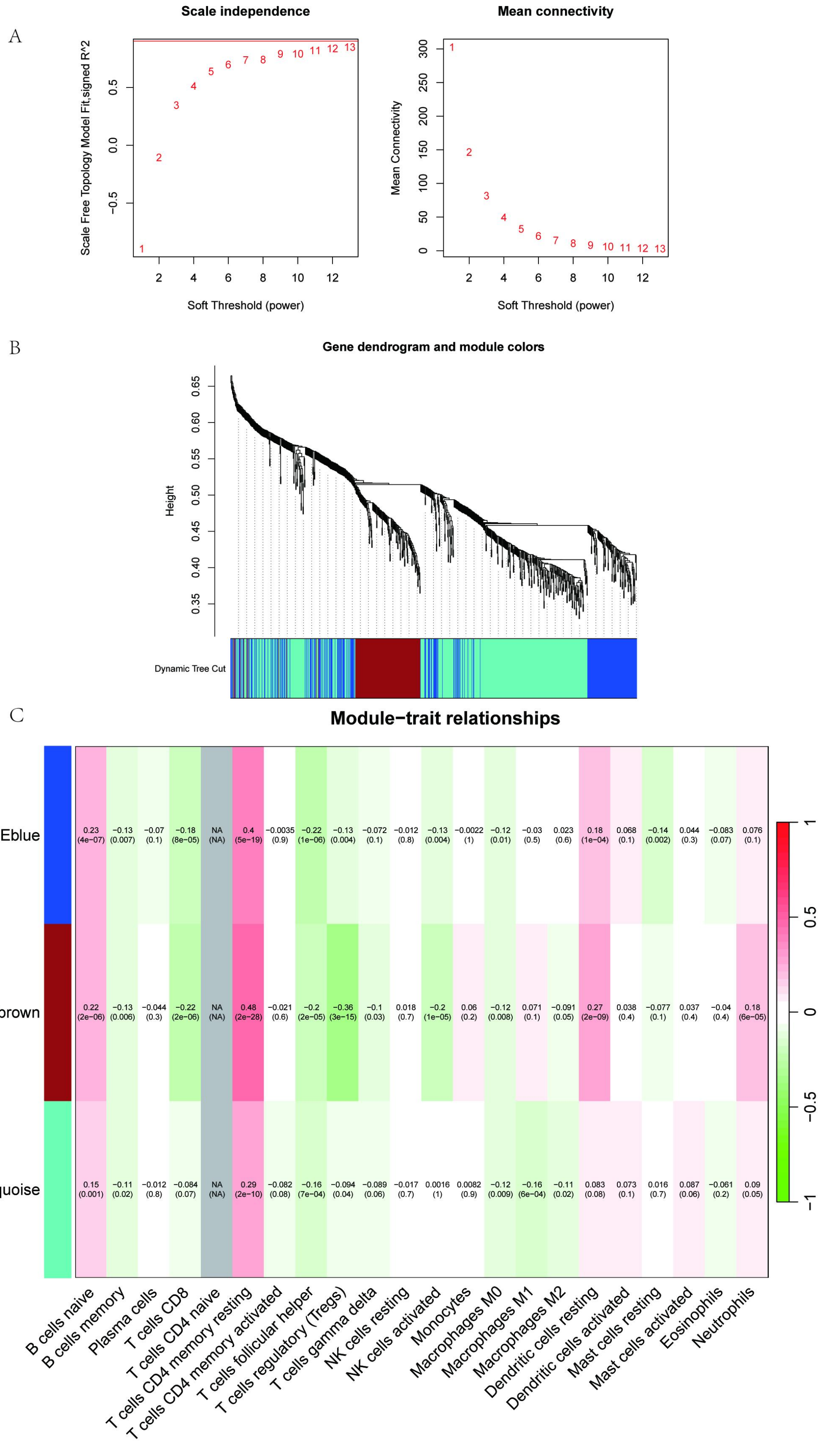

Figure S1. DRGs modules that regulate or are regulated by immune cells. (A) Analysis of the scale-free fit index and average connectivity for various soft-thresholding powers. (B) Dendrogram of module clustering based on dissimilarity measure (1-TOM). (D) Heatmap illustrating correlations between module DRGs and immune cell scores.
